# A Case of Problem-Free Survival Five Years After Abdominal Aortic Sigmoid Colon Fistula Surgery

**DOI:** 10.7759/cureus.75026

**Published:** 2024-12-03

**Authors:** Tomohiro Nakajima, Tsuyoshi Shibata, Shuhei Miura, Yutaka Iba, Nobuyoshi Kawaharada

**Affiliations:** 1 Cardiovascular Surgery, Sapporo Medical University, Sapporo, JPN

**Keywords:** colon bleeding, infection, omentopexy, secondary aortoenteric fistula, stoma

## Abstract

An a*ortoenteric fistula* (AEF) is a rare but life-threatening condition where an abnormal connection forms between the aorta and the gastrointestinal tract, most commonly the duodenum. It can be primary (arising spontaneously due to an aortic aneurysm or infection) or secondary (complicating prior vascular surgery). Immediate recognition and surgical intervention are critical to manage severe gastrointestinal bleeding and prevent fatal outcomes. A 71-year-old male developed an AEF following abdominal aortic graft surgery. Four months postoperatively, he presented with persistent lower gastrointestinal bleeding and was diagnosed with a secondary AEF. A staged surgical approach was employed, involving initial bowel resection, stoma creation, and graft cleaning, followed by subsequent aortic graft replacement with omental flap coverage. Postoperatively, a graft rupture at the proximal anastomosis required emergency endovascular stenting, which stabilized the patient. Antibiotic therapy successfully managed graft infection, including meropenem, linezolid, and later levofloxacin. The patient was discharged home on day 65 and has remained free of aortic events for five years. This case illustrates the complexity of AEF management, emphasizing the importance of a multidisciplinary, staged approach to achieve infection control, hemostasis, and long-term stability. He has been followed up in an outpatient clinic since then. He is now 76 years old, five years later, and is progressing without an aortic event.

## Introduction

Aortoenteric fistula (AEF) is a rare but life-threatening complication following abdominal aortic aneurysm (AAA) repair [1]. It can occur as either a primary AEF originating from an untreated aortic aneurysm eroding into the gastrointestinal tract or a secondary AEF developing after surgical or endovascular aortic intervention. Secondary AEF is most commonly associated with graft infection and typically presents with gastrointestinal bleeding, sepsis, or abdominal pain. Although advances in surgical techniques and graft materials have reduced the overall incidence of AEF, its occurrence remains a significant challenge due to its high morbidity and mortality rates.

The pathogenesis of secondary AEF involves graft infection, inflammation, or erosion into adjacent bowel segments, most frequently the duodenum, but can also include the jejunum, ileum, or colon [2]. The condition is rare, with reported incidence rates ranging from 0.5% to 2% in patients undergoing open aortic surgery. However, its true incidence may be underreported due to difficulties in diagnosis and variability in clinical presentation.

The management of secondary AEF is complex and requires a multidisciplinary approach. The goals are infection control, vascular integrity restoration, and prevention of recurrent fistula formation. Surgical strategies often involve staged procedures, including bowel resection, removal of the infected graft, in situ or extra-anatomic vascular reconstruction, and aggressive antimicrobial therapy. Endovascular techniques have emerged as an adjunct or bridge to definitive surgery in hemodynamically unstable patients [3].

This report describes a case of secondary AEF following abdominal aortic graft placement, managed with a staged surgical approach [4]. The challenges encountered and the long-term favorable outcome achieved underscore the importance of individualized treatment strategies for this rare but serious condition.

## Case presentation

**Figure 1:**
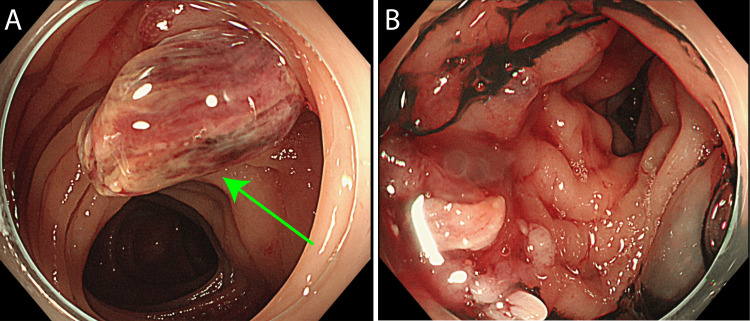
Endoscopic Findings at the previous hospital (A) Fistula and hemorrhage in the sigmoid colon (Green arrow); (B) Hemostasis was obtained by spraying thrombin against the bleeding site.

A 71-year-old male underwent open surgical abdominal aortic aneurysm repair at another hospital using an 18×9 mm J Graft Shield Neo (Japan Lifeline Co., Ltd., Tokyo, Japan). Postoperative inflammation persisted, necessitating prolonged antibiotic therapy. Four months later, the patient presented with lower gastrointestinal bleeding. A colonoscopy revealed a diverticular depression with active bleeding in the sigmoid colon, which was difficult to control via clipping; thrombin was applied to achieve hemostasis (Figure 1). Suspecting an aortocolonic fistula, the patient was transferred to our hospital via emergency services.

**Figure 2:**
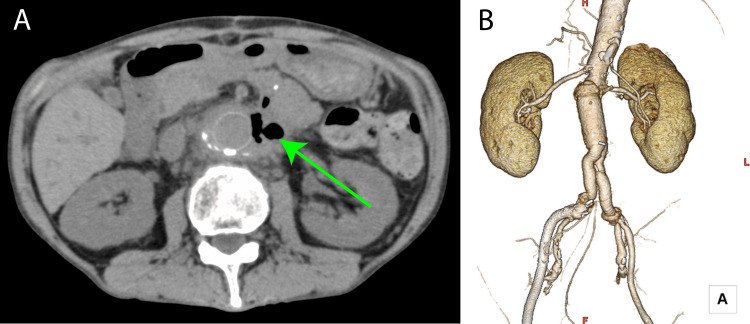
Preoperative enhanced computed tomography (A) Horizontal section image. The air around the artificial vessel was observed. (green arrow); (B) Volume rendering image. The abdominal aorta had been replaced by an artificial vessel by the previous physician.

CT imaging revealed air surrounding the graft, confirming secondary AEF (Figure 2). A two-stage surgical strategy was planned. The first stage involved resectioning the sigmoid colon, stoma creation, and graft irrigation. Two days later, the second stage involved the removal of the infected graft and replacement with an 18×9 mm Gelsoft Plus graft (Terumo Aortic, Glasgow, Scotland), with concurrent omental coverage. Adhesions at the proximal anastomosis required partial retention of the previous graft and felt material. Cultures from the explanted graft identified Bacteroides fragilis, initiating meropenem (MEPM) and linezolid (LZD) treatment.

**Figure 3:**
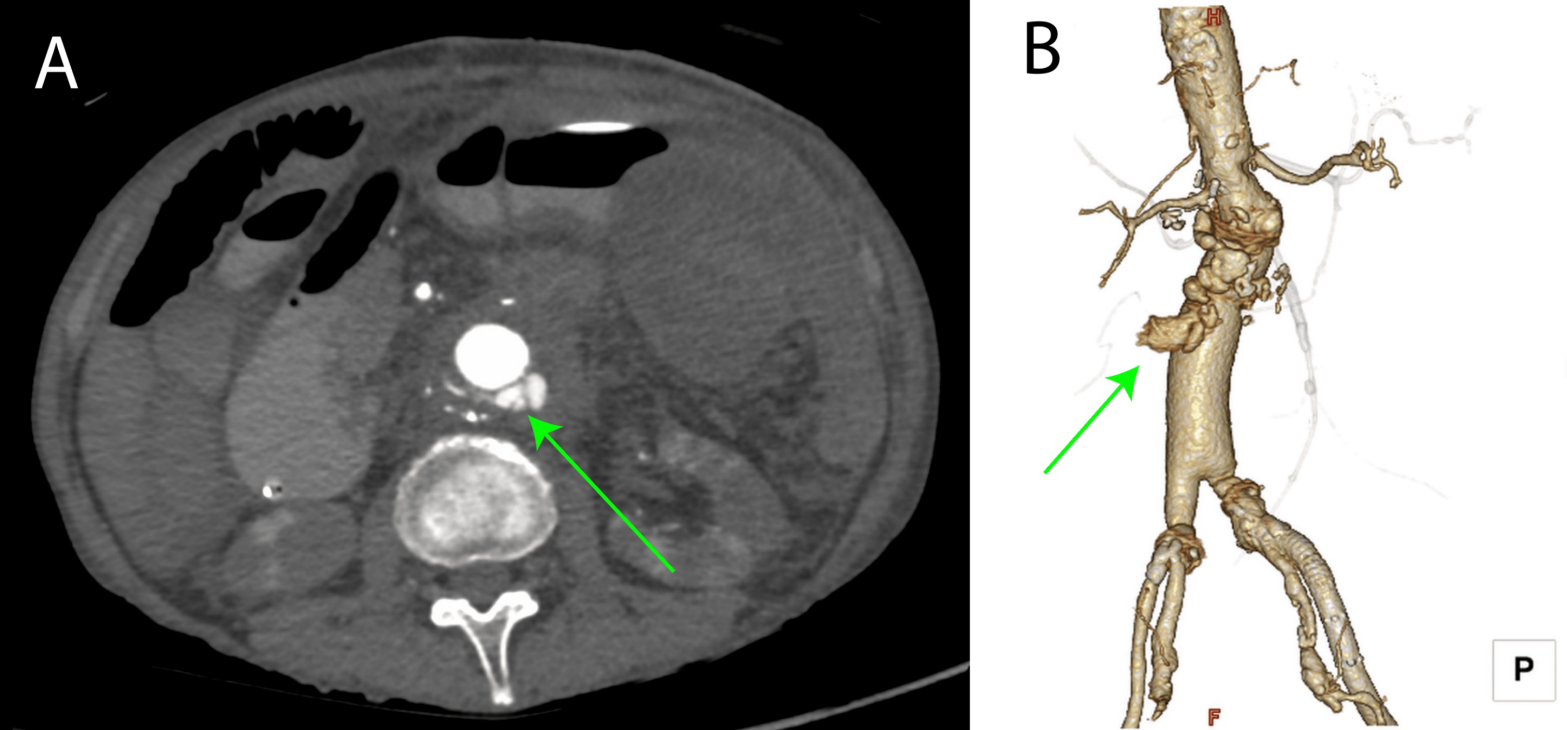
Postoperative enhanced computed tomography (A) Horizontal section image. Bleeding from the central artificial vascular anastomosis was observed, bleeding dorsally. (green arrow); (B) Volume rendering image. The dorsal view showed hemorrhage. (green arrow).

**Figure 4:**
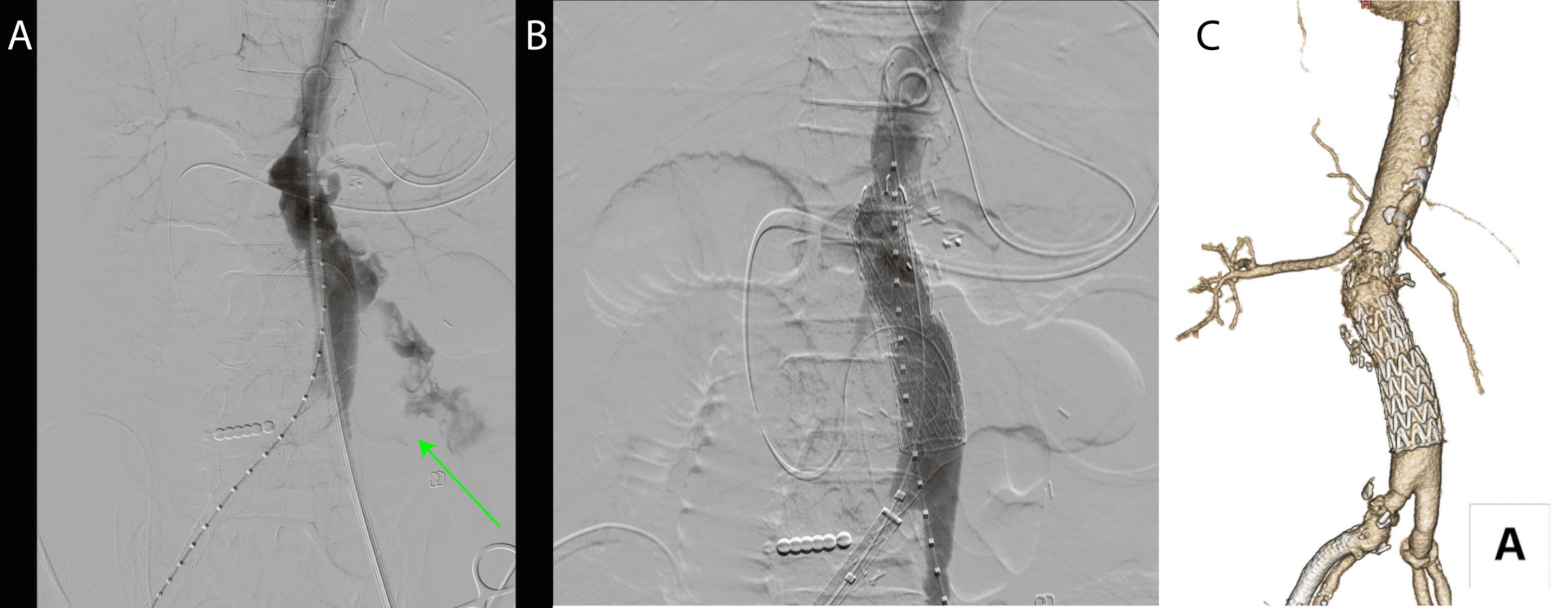
Intraoperative images during endovascular treatment of central hemorrhage (A) Bleeding from the central side of the abdominal aortic prosthesis anastomosis was observed. (green arrow); (B) Stent grafts provided hemostasis, and no bleeding was observed. (C) Enhanced computed tomography. A stent graft covers the central anastomosis.

**Figure 5:**
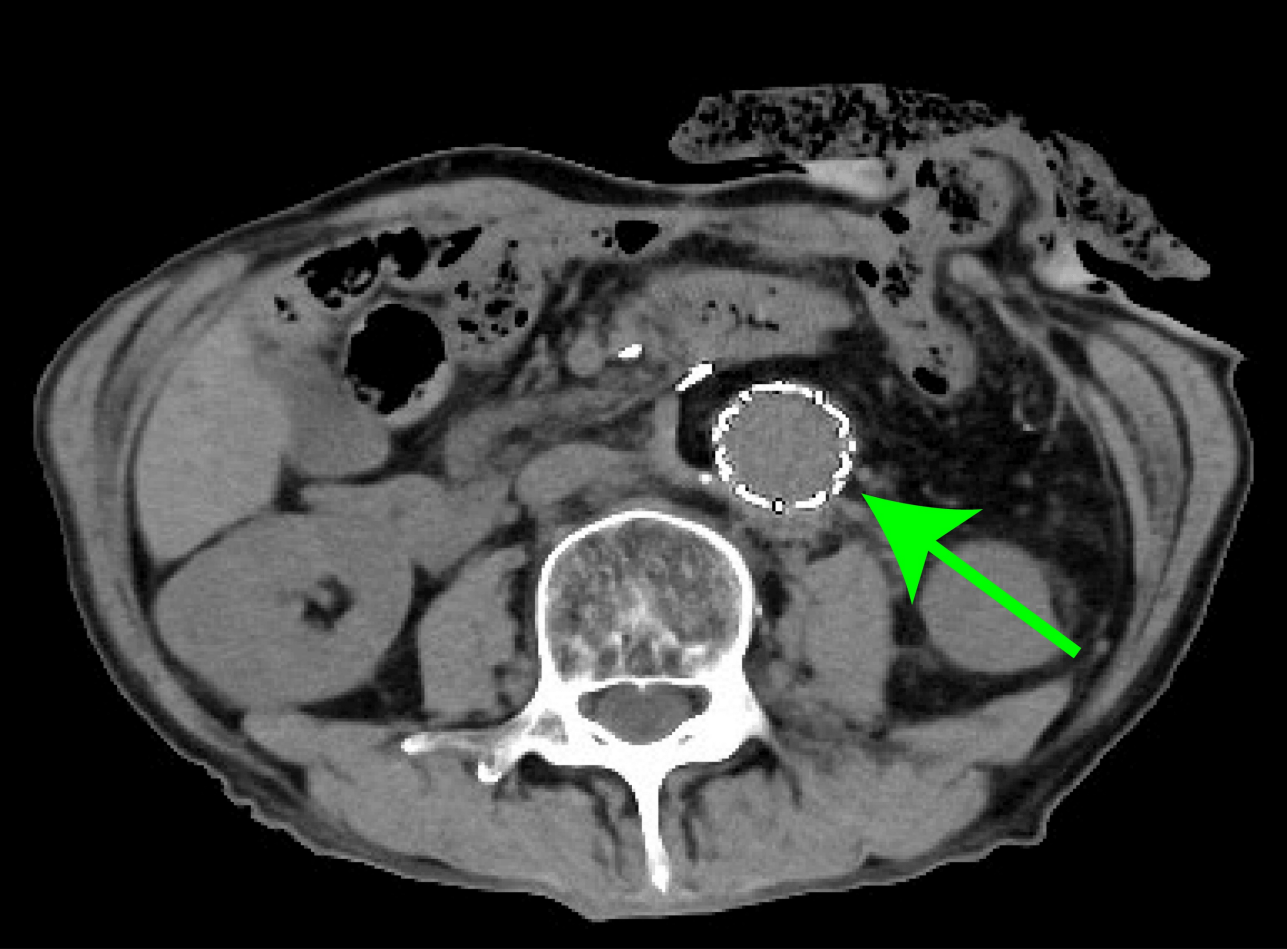
CT image at five-years post-operatively A stent graft was observed to be inserted into the artificial blood vessel. Surrounding the graft, the omentum was packed to provide reinforcement and promote infection control.

On postoperative day five, the patient developed hemodynamic instability. CT imaging revealed proximal anastomotic rupture (Figure 3). Emergency endovascular repair was performed using a 26mm Conformable Thoracic Aortic Graft (cTAG; W. L. Gore & Associates, Flagstaff, AZ, USA) and an additional 23mm Excluder AAA Endoprosthesis (W. L. Gore & Associates, Flagstaff, AZ, USA) cuff for hemostasis (Figure 4). Subsequent recovery was uneventful, with antibiotics switched to levofloxacin (LVFX) by day 55. The patient was discharged home on day 65 without recurrent infection. At the five-year follow-up, now aged 76, the patient remains free from aortic events (Figure 5).

## Discussion

Secondary AEF is a rare but severe complication of vascular graft surgery, carrying a high mortality risk. This case highlights the challenges in managing secondary AEF, particularly in the context of infection and proximal anastomotic rupture. Early detection and multidisciplinary planning are crucial for optimizing outcomes [5].

Secondary AEF typically occurs months to years after primary aortic surgery and often presents with gastrointestinal bleeding or sepsis. Diagnosis remains challenging; imaging, such as CT, often plays a pivotal role in identifying hallmark signs like perigraft air or fistulous connections. The surgical approach, in this case, a two-stage procedure involving graft replacement and omental coverage, represents a well-established strategy for managing infected grafts while minimizing perioperative risks [6].

Endovascular stent grafting, used emergently in this case, has increasingly become a valuable adjunct in secondary AEF management. While traditionally considered a bridge to definitive open repair, stent grafting may offer durable outcomes in carefully selected patients, particularly when complete graft excision poses excessive risk. Using a conformable thoracic aortic graft (cTAG) and an excluder cuff effectively controlled bleeding from the proximal anastomotic rupture, underscoring the utility of advanced endovascular techniques in critical situations [7].

Infection control is a cornerstone of secondary AEF management. Cultures from the explanted graft confirmed Bacteroides fragilis, guiding targeted antibiotic therapy. Long-term suppression with levofloxacin likely contributed to the patient’s favorable 5-year outcome, as chronic infection control remains critical for graft preservation and overall survival [8].

Despite successful treatment, secondary AEF highlights the limitations of current graft materials and surgical techniques. Novel approaches, including antibiotic-impregnated grafts or bioengineered vascular prostheses, are under investigation and promise to reduce infection rates and improve long-term durability.

This case emphasizes the importance of individualized care in secondary AEF management, balancing surgical complexity, infection control, and patient comorbidities. The patient’s uneventful 5-year follow-up demonstrates the potential for excellent outcomes, even in the face of severe complications, when appropriate surgical and medical strategies are employed.

## Conclusions

The patient, a 71-year-old male, developed a secondary AEF four months after undergoing open abdominal aortic aneurysm repair. After presenting with hematochezia and signs of infection, a staged surgical approach was performed, including sigmoid colon resection and graft replacement using a new graft and omentopexy. Despite postoperative complications, including central anastomotic rupture requiring endovascular stent graft repair, the patient recovered and has remained event-free for five years. This case highlights the complexity and challenges in managing secondary AEFs.
